# Ultra-Processed Foods in Early Life: Biological Mechanisms, Developmental Programming and Childhood Obesity—A Narrative Review

**DOI:** 10.3390/nu18193214

**Published:** 2026-09-29

**Authors:** Oana-Raluca Temneanu, Laura Iulia Bozomitu, Adriana Mihai, Andreea-Luciana Avasiloaiei, Vasile Valeriu Lupu, Otilia Novac, Felicia Trofin, Ancuta Lupu, Mihaela Monica Alexoae, Bogdan Novac, Emil Anton, Elena Hanganu, Raluca Olariu, Ileana Katerina Ioniuc

**Affiliations:** Grigore T. Popa University of Medicine and Pharmacy, 700115 Iași, Romania; temneanu.oana@umfiasi.ro (O.-R.T.); andreea.avasiloaiei@umfiasi.ro (A.-L.A.); vasile.lupu@umfiasi.ro (V.V.L.); otilia.novac@umfiasi.ro (O.N.); felicia.trofin@umfiasi.ro (F.T.); ancuta.ignat1@umfiasi.ro (A.L.); mihaela.alexoae@umfiasi.ro (M.M.A.); bogdan.novac@umfiasi.ro (B.N.); emil.anton@umfiasi.ro (E.A.); hanganu.elena1@umfiasi.ro (E.H.); raluca.olariu@umfiasi.ro (R.O.); ileana.ioniuc@umfiasi.ro (I.K.I.)

**Keywords:** ultra-processed foods, NOVA classification, early-life programming, first 1000 days, childhood obesity, gut microbiota, intestinal permeability, complementary feeding

## Abstract

Childhood obesity now affects more than 160 million children and adolescents worldwide, and over the same period ultra-processed foods have come to dominate the early-life diet. This review sets out a single argument: that the effect of ultra-processed foods on childhood obesity is better read developmentally, and that the digestive tract is the organ through which much of that effect is transmitted. Exposure begins before birth and continues through the first 1000 days and complementary feeding, the period in which the gut microbiota assembles and the intestinal barrier matures. We show how ultra-processed foods perturb these two systems in particular: a low-fibre, additive-rich, energy-dense diet reshapes microbial community structure and depletes short-chain fatty acid production, while emulsifiers and other additives thin the mucus layer and raise intestinal permeability, admitting microbial products that sustain low-grade inflammation. We trace how these gut-level changes feed forward into altered appetite regulation, adipocyte biology and, ultimately, excess adiposity, and we situate the other proposed mechanisms (matrix disruption, hyperpalatability, accelerated eating rate, endocrine-disrupting additives and central reward signalling) around this digestive core, marking at each step where the evidence is paediatric and where it rests on adult or animal data. The epidemiological literature is appraised by study design, and the constraints on causal inference are stated plainly: NOVA misclassification, residual confounding, reverse causation, dietary misreporting and the scarcity of paediatric trials. The practical implication is that prevention framed around early-life gut health, rather than around cumulative adult-style exposure, is where intervention is most plausibly effective. We are explicit throughout that most of the mechanistic evidence derives from animal or adult studies and that a causal role for ultra-processed foods in childhood adiposity, though biologically plausible, is not yet established in paediatric populations; the developmental reading we advance is the most coherent interpretation of an associational evidence base, not a demonstrated causal chain.

## 1. Introduction

Childhood obesity has increased dramatically over the last three decades, and more than 160 million children and adolescents worldwide now live with the condition [1]. It is not a state that resolves with age. Adiposity tracks forward across the lifespan with correlation coefficients clustering around 0.6, so that a child with obesity carries a substantially elevated probability of becoming an adult with obesity and its cardiometabolic sequelae [2]; the association between childhood adiposity and adult cardiovascular risk appears to be mediated largely by this persistence of excess weight rather than by an effect independent of it [3]. Childhood obesity itself carries metabolic and cardiovascular risk that is not fully deferred to adulthood, since type 2 diabetes, hypertension, and dyslipidaemia are now documented in children and adolescents with obesity [4,5].

Over the same period, the composition of the child’s diet has changed. The change is one of composition as much as of processing: the products that have grown to dominate children’s diets are typically dense in energy, saturated and industrially modified fats, added sugars including high-fructose corn syrup, and sodium, while being low in fibre and in the micronutrients that whole foods supply. A healthy diet, by contrast, is built on minimally processed foods—vegetables and fruit, whole grains, legumes, fish, dairy and water—and it is against this reference pattern that the ultra-processed diet is most usefully judged. Ultra-processed foods now supply a large fraction of energy intake in many countries, and rising sales of these products have co-occurred with increasing population body-mass index across nations [6,7]. Among infants and toddlers, ultra-processed items already contribute a substantial share of energy from complementary foods and beverages, once human milk and formula are set aside [8]. By early childhood the contribution is larger still: recent syntheses of United States intake report that toddlers and school-aged children obtain approximately 47% and 59% of their daily energy, respectively, from ultra-processed foods [9]. The coincidence is striking. It is also, on its own, uninformative: rising sales track many other things, and the central question this review addresses is whether, how, and above all when ultra-processed food exposure exerts a causal influence on the development of obesity.

That question of timing is what distinguishes paediatric nutrition from adult nutrition, and it organises what follows. The developing organism passes through periods of heightened plasticity during which environmental inputs can produce structural and regulatory changes that persist long after the exposure ends. An exposure that is metabolically trivial in a forty-year-old may be formative in a two-year-old, because it arrives while the systems that will regulate energy balance for a lifetime are still being assembled. Under the developmental origins of health and disease framework, the periconceptional period, gestation, infancy, and early childhood are not simply earlier points on a dose–response curve; they are windows in which the curve itself is drawn [10].

A definitional clarification is required before proceeding, because the field’s central disputes are largely definitional. “Ultra-processed” is not a synonym for energy-dense, sugary, or nutrient-poor, although these properties frequently co-occur. The NOVA classification, which underpins most of the literature reviewed here, sorts foods by the nature and purpose of industrial processing rather than by nutrient content, dividing all items into four groups; the fourth, the ultra-processed group, comprises industrial formulations made predominantly from substances extracted from foods, combined with cosmetic and functional additives, and containing little or no intact whole food [11,12]. A reduced-sugar fortified cereal and a sugar-sweetened one may share a NOVA group despite divergent nutrient profiles. This decoupling of processing from composition frames the first of two central questions structuring this review: if ultra-processed foods affect metabolic health partly through attributes other than their macronutrient content, nutrient-based dietary guidance will systematically under-address the problem.

The second question is the one of timing, and it supplies the review’s organising axis and its central objective: to ask whether ultra-processed food exposure during the critical developmental windows of early life contributes to childhood adiposity, and what strength of evidence—and specifically what paediatric evidence—supports a causal rather than a merely associational reading. We trace a single developmental chain (prenatal nutrition, breastfeeding, complementary feeding, ultra-processed exposure, microbiota, inflammation, immune maturation, epigenetic change, adiposity, and adult disease) and ask, at each link, what the evidence supports and what it does not. We first examine the exposure and the measurement problems specific to paediatric research, including whether the NOVA system is adequate to the task at all. We then set out the developmental windows and the biology that makes them windows. Against that background we consider eight biological mechanisms by which ultra-processed foods may act, flagging throughout where the evidence is paediatric and where it is extrapolated from adults or animals. We appraise the epidemiological evidence by study design, confront its limitations candidly, and close with confront its limitations candidly, and close with the implications for paediatric practice and public-health policy, together with the research questions whose answers would move the field from association towards causation.

## 2. Defining the Exposure: The NOVA Classification

The NOVA system sorts foods into four groups according to the extent and purpose of industrial processing: unprocessed or minimally processed foods, processed culinary ingredients, processed foods, and ultra-processed foods [11,12]. The fourth group is the object of concern here. Its members are industrial formulations containing little or no intact whole food, assembled from refined substrates such as starches, isolated proteins, sugars, and oils, together with additives serving cosmetic or functional ends, such as flavours, colours, emulsifiers, and non-nutritive sweeteners [11]. Developed originally in Brazil, the system has since been adopted to varying degrees by national dietary guidelines and international agencies [12], and standardised protocols now exist for applying it to survey data [13,14].

Its principal strength is also its principal vulnerability. By classifying on processing rather than composition, NOVA captures something that nutrient profiling misses, but it does so at the cost of internal heterogeneity and, at times, of reproducibility. When approximately 170 French food and nutrition specialists independently assigned foods to NOVA groups, agreement was poor, with Fleiss’ kappa reaching only 0.32 for marketed products and 0.34 for generic items, values indicating no better than fair concordance [15]. Independent appraisals have echoed this concern, noting that a non-trivial fraction of foods routinely labelled ultra-processed have acceptable nutritional quality, and that the category groups together items of very different likely effect [16,17].

### 2.1. Is NOVA Sufficient for Paediatric Research?

These classification difficulties are compounded in the paediatric setting. Five features of the paediatric diet expose the limits of the classification with particular clarity, and taken together they raise the question of whether NOVA, as currently applied, is adequate to paediatric research at all.

The first, and by some distance the most delicate, is infant formula. Whether formula should be counted as ultra-processed depends on how the NOVA framework is applied, and practice varies. Some analyses place it in the ultra-processed group on the basis of its manufacture, since it is assembled industrially from refined constituents such as milk proteins, vegetable oils, lactose, micronutrients and added ingredients [18]; others treat it as a special case that sits uneasily within a system designed to describe discretionary foods [19]. The distinction matters less than what it obscures. NOVA classifies by the nature of processing, not by nutritional value, safety, or clinical necessity, and on each of these last three counts, infant formula stands apart from the discretionary products with which a processing-based label would group it. For an infant who is not breastfed, formula is a nutritionally complete food and the only safe alternative to human milk. Its classification under NOVA, whatever position one takes, is therefore a statement about manufacturing rather than about the appropriateness of the food, and treating the two as equivalent, in either direction, would be a category error.

The consequences for inference are not hypothetical. Studies that include formula in the ultra-processed category will find ultra-processed intake associated with formula feeding, and formula feeding associated with growth outcomes, without any of this establishing an effect of processing; in such analyses the exposure variable reflects feeding mode as much as food processing. The problem is compounded by the social patterning of formula use, which correlates with maternal employment, education, and access to lactation support, the same variables that independently predict childhood adiposity.

The difficulty here is an instance of a more general objection to NOVA that has crystallised into open scientific dispute. Critics observe that the classification sorts by processing history rather than by any calibrated dose of a harmful constituent, so that products with radically different nutritional profiles and plausible effects share a category; as one widely cited formulation of the objection has it, soya burgers are not confectionery, even though NOVA classifies them alike [20]. Louie has pressed the argument further, contending that NOVA exhibits an internal logical incoherence and sits uneasily with the dose-dependent reasoning that governs the rest of nutritional toxicology [21], and has called for greater methodological rigour in a field where, in his account, methodological critique is too often dismissed by rhetorical rather than scientific means [22]. Proponents of the classification reply that the heterogeneity of ultra-processed foods is typical of dietary exposures generally, that the category’s value lies in its consistent prediction of harm at the level of the whole diet and its capacity to inform policy, and that persistent scepticism about the evidence base delays public health action [23]. Two perspectives setting out these opposing positions were published alongside one another in Nature Food in 2026, an unusual editorial acknowledgement that the scientific community is genuinely divided [24].

We take no side in that dispute here, but its paediatric implications are direct. If NOVA cannot distinguish formula from confectionery, then paediatric studies that report “ultra-processed intake” in infants are reporting a composite whose interpretation depends entirely on how much of it is formula. Some authors have accordingly argued that the infant sector requires its own classificatory and regulatory framework, distinct from that applied to the general packaged food supply [18,25]. The regulatory landscape reinforces the case: the WHO International Code of Marketing of Breast-Milk Substitutes, adopted in 1981, restricts the marketing of formula directed at infants under twelve months, but growing-up formulas aimed at children aged twelve to thirty-six months and packaged baby foods fall outside its scope, a gap into which manufacturers have expanded, in a global infant and toddler feeding market that grew from around 33 to 68 billion United States dollars between 2010 and 2022, with growing-up formula the fastest-expanding segment [18]. The commercial interest in an ambiguous category is therefore considerable, and it cuts in both directions: industry has reason to resist the ultra-processed designation, and advocates have reason to apply it broadly. Neither motive is an argument.

Our own position is narrow and, we hope, uncontroversial. Formula should be reported separately from discretionary ultra-processed products in paediatric research, not because it is beyond criticism, but because pooling them makes the resulting effect estimate uninterpretable. Where studies do not disaggregate, their findings on infants should be read with this limitation in view.

The second is commercial baby foods, and here the evidence pulls in two directions that a narrative review is obliged to report rather than reconcile. Purées, pouches, infant cereals, and snack products marketed for weaning span a wide range of formulations, and their assessment has produced genuinely divergent emphases in the literature. On one side, several product audits document a category dominated by processing and additives. In an assessment of commercial infant foods in Brazil, around 79% met ultra-processed criteria, the largest categories being breast-milk substitutes and cereals [26]. In a cross-sectional analysis of 651 infant and toddler products sold by major United States retailers, additives such as thickeners and emulsifiers were the dominant reason for ultra-processed classification, accounting for roughly 71% of classifications, and these products carried more sugar and sodium than their non-ultra-processed counterparts [19]. Squeeze pouches attract particular scrutiny: an audit of 276 Australian products found that only two met a nutrient-profiling standard, that 59% carried a ‘no added sugar’ claim despite containing free sugars, and that sugar content reached 17.5 g per 100 g, with most products not fortified with iron [27]. European surveys reach broadly concordant conclusions [28]. On the other side, paediatric nutrition bodies have cautioned against a blanket condemnation. The Committee on Nutrition of the French Society of Paediatrics judged that commercial baby foods offer real advantages in microbiological safety, that their nutritional merit varies from product to product rather than uniformly, and that advising parents as though every commercial option were a poor choice is neither accurate nor helpful [29]. Narrative reviews of commercially prepared infant foods in high-income countries reach a similarly qualified verdict, noting adequate provision of some nutrients alongside excesses of others [30]. These products are, moreover, defined and constrained by regulation (in the European Union by Directive 2006/125/EC, with compositional and hygiene criteria that ordinary packaged foods need not meet), so that the NOVA label and the regulatory status describe different properties of the same product. The reasonable reading is not that one side is right. It is that the ultra-processed designation, applied to this category, aggregates a genuinely heterogeneous set of products whose nutritional quality ranges from poor to acceptable, and that a classification driven largely by the presence of a thickener does not, on its own, identify a harmful food. This is precisely why disaggregation matters more here than almost anywhere else in the ultra-processed literature.

The third is texture. Paediatric feeding involves a deliberate progression of textures, from liquid through purée to solids, chosen for developmental rather than commercial reasons. NOVA is blind to texture, yet texture is among the more plausible mediators of the effect of processing on energy intake, as Section 4 discusses. A soft, homogenised food may promote passive overconsumption whether it is a commercial pouch or a home-prepared purée.

The fourth is the food matrix. NOVA classifies by ingredient list and processing history, not by the physical structure in which nutrients are embedded. Two products with identical NOVA classification may differ substantially in matrix integrity, and matrix integrity governs digestion rate, glycaemic response, and satiation.

The fifth is additives. Within group 4, additive burden varies by an order of magnitude. Emulsifiers, of specific concern for the developing gut, are present in some ultra-processed foods and absent from others; the classification does not distinguish them.

### 2.2. Compositional Concerns Beyond Processing

Because NOVA classifies on processing rather than composition, the components that most plausibly mediate harm are not part of its definition, and they deserve explicit statement. The products that dominate children’s ultra-processed intake tend to share a compositional signature: a high energy density, often exceeding 400 kcal per 100 g in the case of energy-dense snacks and confectionery, against roughly 60–80 kcal per 100 g for most vegetables and fruit; a high content of saturated and industrially modified fats; added sugars, prominently high-fructose corn syrup and sucrose, that raise energy density and, in the case of free fructose, act through the hepatic pathway set out in Section 4.9; elevated sodium; and a correspondingly low content of dietary fibre and intrinsic micronutrients. It is this profile, rather than processing as such, that links the products to adiposity, and it is why two foods sharing a NOVA-4 label can differ so markedly in likely effect.

The distinction from healthy foods follows directly. A food may be minimally processed and yet energy-dense and sugary, and a food may be industrially processed and yet nutritionally useful, as the fortified and wholegrain examples of Section 3 show. The useful contrast in paediatric counselling is therefore not processed versus unprocessed but nutrient-dense whole or minimally processed foods—vegetables, fruit, legumes, wholegrains, fish, eggs, plain dairy and water—set against energy-dense, sugar-rich, fibre-poor discretionary products, whether or not the latter are technically ultra-processed.

None of this argues for discarding NOVA, which remains the best available operationalisation of a real and otherwise unmeasured exposure. It argues that effect estimates built upon it should be interpreted with corresponding caution, and that paediatric studies would benefit from reporting additive burden, matrix integrity, and texture alongside NOVA group, or from disaggregating the category into its constituent products. In hospital paediatric practice, the same distinction becomes relevant when counselling families: advising a parent to reduce “ultra-processed” intake is actionable only if the clinician can specify which items carry the risk and which are benign or necessary.

### 2.3. Materials and Methods

A word on how the evidence discussed below was assembled. This is a narrative review, and it is reported in line with the SANRA (Scale for the Assessment of Narrative Review Articles) criteria for justification of the article’s importance, statement of aims, description of the literature search, referencing, and level of evidence. It makes no claim to the exhaustive, protocol-driven coverage of a systematic review. We searched MEDLINE (via PubMed) and Scopus from database inception to 31 January 2026, with no language restriction. The search combined terms for the exposure (“ultra-processed food*”, “NOVA”, “food processing”) with terms for the population (“infant*”, “child*”, “adolescen*”, “paediatric”, “pregnan*”, “maternal”), the outcome (“obesity”, “overweight”, “adiposity”, “body mass”), and the candidate mechanisms and developmental windows (“gut microbiota”, “intestinal permeability”, “short-chain fatty acid*”, “emulsifier*”, “first 1000 days”, “developmental programming”). The full Boolean search string, adapted to each database, was of the form: (“ultra-processed food*” OR “NOVA” OR “food processing”) AND (“infant*” OR “child*” OR “adolescen*” OR “paediatric” OR “pregnan*” OR “maternal”) AND (“obesity” OR “overweight” OR “adiposity” OR “body mass” OR “gut microbiota” OR “intestinal permeability” OR “short-chain fatty acid*” OR “emulsifier*” OR “first 1000 days” OR “developmental programming”), searching titles, abstracts and keywords. As a narrative review, we did not undertake the formal record counting and screening flow of a systematic review. Reference lists of included articles and relevant reviews were hand-searched to identify further sources. Priority was given to controlled human trials, prospective cohorts, and systematic reviews over cross-sectional and mechanistic animal work where both were available. Selection was purposive rather than consecutive: we sought the strongest evidence on each side of the contested points, and we flag throughout where a claim rests on paediatric data and where it is extrapolated from adult or animal studies.

### 2.4. Heterogeneity Within Paediatric Ultra-Processed Foods

The objection that the NOVA framework aggregates products of very different composition is not merely theoretical; it bears directly on how the paediatric association should be read. Four food groups that contribute substantially to ultra-processed intake in children illustrate the point, because the evidence for each departs from the pattern seen for sugar-sweetened beverages and processed meats.

Breakfast cereals occupy an ambiguous position. Ready-to-eat cereals are classified as ultra-processed, yet higher-fibre and wholegrain varieties are, in several paediatric datasets, among the larger contributors to whole-grain and micronutrient intake rather than markers of a poor diet. In the UK Gemini twin cohort, higher-fibre breakfast cereals were a principal ultra-processed subgroup in toddlerhood, giving way to sweetened cereal products only in mid-childhood [31]. Whether a cereal behaves as a risk food or a fibre source therefore depends on the specific product, not on its NOVA group.

Yogurts and fermented dairy present the same difficulty in sharper form. Many commercial children’s yogurts carry the additives and added sugars that place them in the ultra-processed category, yet the food matrix delivers protein, calcium and live cultures, and fermented dairy tends to be associated with neutral or favourable cardiometabolic profiles in children rather than with adiposity. Analyses that separate ultra-processed subgroups bear this out: in a Swedish cohort of eight-year-olds, higher intake of ultra-processed beverages tracked with an adverse inflammatory profile, whereas the bread-and-cereal and protein subgroups showed no such association [32].

Fortified foods complicate the picture further, since fortification is often the mechanism by which an industrially produced food delivers iron, vitamin D or B-vitamins to children who would otherwise fall short. To treat a fortified product as equivalent, on processing grounds alone, to a confectionery item is to disregard the very nutrients that justify its place in the diet.

Wholegrain and packaged breads make the contradiction explicit. Mass-produced sliced bread is ultra-processed by NOVA criteria, yet it is, in several countries, among the largest contributors to children’s whole-grain intake, and wholegrain items are consistently associated with reduced rather than increased cardiometabolic risk [32]. A blanket instruction to avoid ultra-processed foods would, taken literally, lower whole-grain consumption.

These divergences bear on a question the epidemiology rarely poses explicitly: which ultra-processed categories drive the association with adiposity? Where studies have disaggregated the exposure, the signal concentrates in sugar-sweetened beverages, confectionery, energy-dense savoury snacks and processed meats, while breads, cereals and dairy contribute little or move in the opposite direction [33]. In one analysis of children and adolescents, total ultra-processed intake was not associated with overweight or obesity once the contributing groups were examined separately, the association being carried by specific discretionary items rather than by the category as a whole [33]. Treating ultra-processed food as a single homogeneous exposure therefore risks averaging genuinely harmful products together with neutral or beneficial ones, attenuating the very effect that motivates concern.

At the opposite end of the heterogeneity spectrum sit the energy-dense fast foods that anchor the harmful pole of the ultra-processed category. French fries, fried chicken, burgers, doughnuts and the sugar-sweetened drinks that accompany them derive most of their energy from fat and refined carbohydrate, and their risk is neither theoretical nor confined to NOVA labelling: in a prospective study of 424 Iranian children and adolescents, those in the highest quartile of fast-food intake had almost three times the incidence of metabolic syndrome over 3.6 years compared with the lowest, together with raised triglycerides and abdominal adiposity [34]. Where the case for some ultra-processed subgroups is genuinely uncertain, the case against habitual fast-food consumption in childhood is not, and it should not be diluted by the ambiguity surrounding breads, cereals or yogurts.

## 3. Early-Life Programming: Why Timing Matters

**Figure 1:**
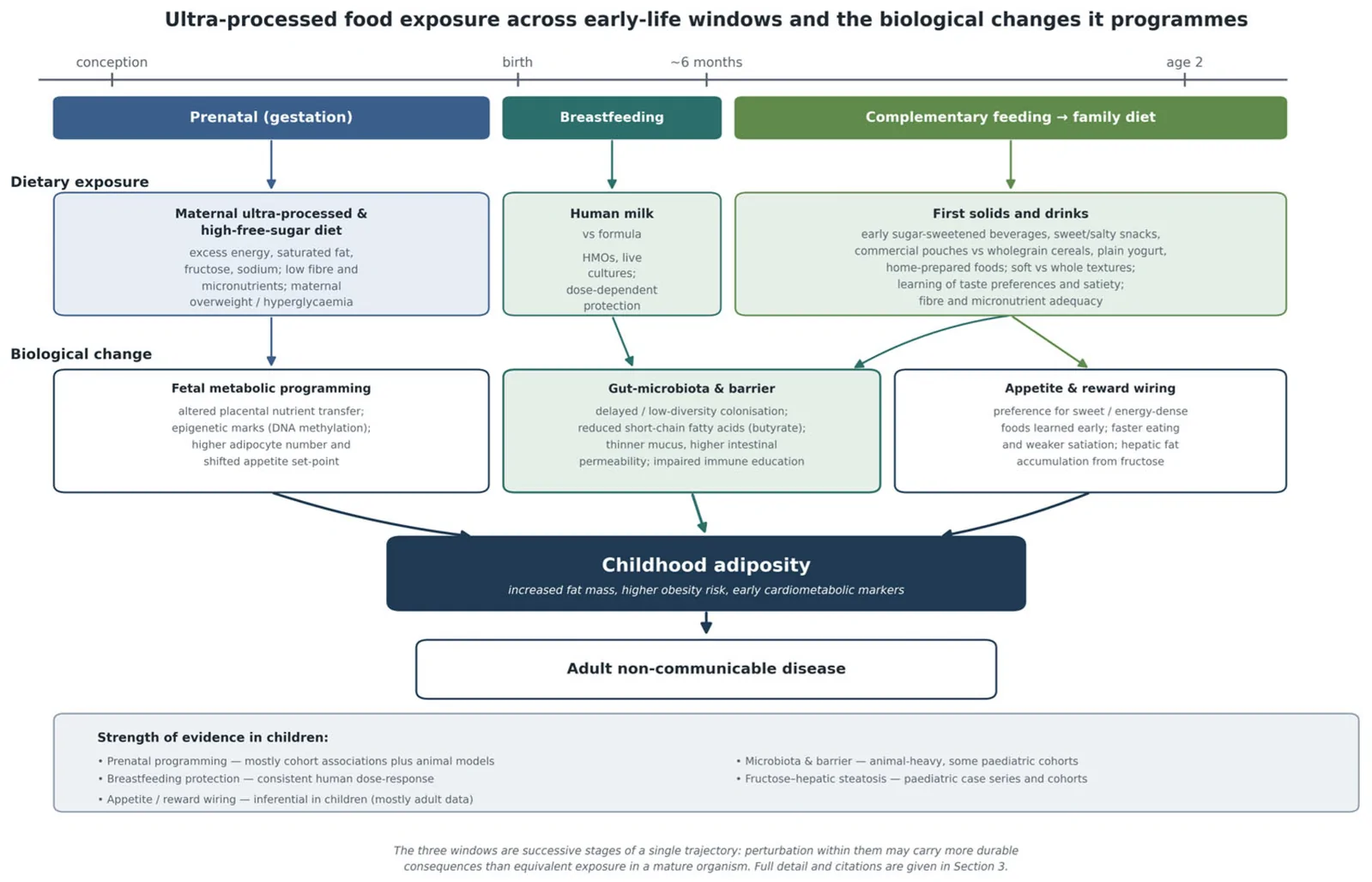
Early-life nutritional programming. For each developmental window—prenatal, breastfeeding, and complementary feeding—the figure shows the principal dietary exposures, the biological changes they programme, and the strength of the supporting evidence in children. Full detail and citations are given in Section 3.

The proposition that adult disease has developmental origins is now well established, and it supplies the framework within which paediatric ultra-processed exposure should be read. Adverse conditions during gestation and early postnatal life induce structural and functional adaptations in developing organs, adaptations that may be advantageous in the anticipated environment and maladaptive in the one actually encountered [10]. Three windows concern us: the prenatal period, the first **1000** days, and the transition to family foods through complementary feeding. They are not separate compartments but successive stages of a single trajectory, and the developmental chain that runs through them is the organising axis of this review. Figure 1 summarises the developmental chain that organises this review.

### 3.1. Prenatal Exposure

**Maternal diet.** The intrauterine environment is shaped by what the mother eats, and maternal ultra-processed intake has been examined as a determinant of offspring adiposity. The largest such study, pooling three United States cohorts comprising 19,958 mother–child pairs, found no significant association between maternal ultra-processed intake during the peripregnancy window itself and offspring overweight or obesity [35]. It was maternal consumption during the postnatal period that carried the association, with a 26% higher risk in the highest versus the lowest category of intake [35]. This dissociation is instructive, and it cuts against an over-eager programming interpretation of the epidemiology: the data are at least as compatible with a shared postnatal feeding environment as with an intrauterine effect, since maternal diet is socially patterned and correlated with the diet the child will later be offered. The mechanistic case for a prenatal effect, however, rests on more than these cohort data.

**Placental programming.** The placenta is not a passive conduit but an active programming agent. Its phenotype adapts to maternal diet and metabolic status, and these adaptations alter fetal nutrient supply in ways that have lasting consequences [36]. Maternal high-fat feeding and maternal obesity modify placental morphology, nutrient transport capacity, and hormonal signalling; the responses show sexual dimorphism, with male and female fetuses differing in the timing and severity of downstream outcomes [36]. Maternal nutritional disorders, whether deficiency or excess, compromise placental efficiency through altered nutrient transport and hormonal signalling, and the resulting perturbations of fetal growth are associated with increased susceptibility to non-communicable disease in adulthood [36].

**Epigenetics.** The mechanism by which a transient prenatal exposure produces a durable phenotype is, in substantial part, epigenetic. Maternal nutrition and toxicant exposures converge on the fetal epigenome, inducing changes in DNA methylation and histone modification at loci governing metabolic regulation [25]. This convergence is directly relevant to ultra-processed foods, which deliver not only a particular macronutrient profile but also endocrine-disrupting chemicals capable of crossing the placenta and altering nuclear receptor signalling and methylation patterns in metabolic tissues [25]. The epigenetic route thus unifies two mechanisms that are often treated separately, the nutritional and the toxicological, and it explains how an exposure confined to gestation can express itself as adiposity decades later. It should be said plainly that the human evidence for this pathway remains largely associational, and that much of the mechanistic work derives from animal models.

### 3.2. The First 1000 Days

The period from conception to a child’s second birthday, conventionally the first 1000 days, is when metabolic, immune, and neuroendocrine systems undergo their most rapid maturation [10,37]. Three developmental processes concentrated in this window are relevant here.

**Microbiota assembly.** The infant gut is colonised rapidly after birth, initially by facultative anaerobes and subsequently by obligate anaerobes including *Bifidobacterium*, *Bacteroides*, and *Clostridium* [37,38]. In breastfed infants the community is dominated by species that metabolise human milk oligosaccharides, and roughly a quarter to a third of the infant bacterial microbiota is thought to originate from breast milk itself [37]. The mode of delivery shapes this initial colonisation. Infants born vaginally are seeded by maternal vaginal and faecal microbiota, acquiring *Lactobacillus*, *Bacteroides*, and *Bifidobacterium*, whereas those born by caesarean section are colonised initially by maternal skin and environmental organisms such as *Staphylococcus*, with delayed acquisition of beneficial taxa and the reduced early diversity that characterises early-life dysbiosis [39]. Whether this difference translates into a durable effect on adiposity is contested: a large contemporary cohort found that the association between caesarean birth and childhood obesity disappeared for elective sections once macrosomic infants were excluded, and persisted only for emergency sections, a pattern difficult to reconcile with a causal role for missing vaginal microbial exposure [40]. Diversity remains narrow through early infancy and expands sharply at weaning, when dietary diversification drives a major compositional shift [38]. Because the community is assembling rather than established, perturbations during this window may carry more durable consequences than equivalent perturbations of a mature adult ecosystem, and early-life dysbiosis has been linked to later obesity, allergic disease, and metabolic disorder [38,41].

**Immune maturation.** Microbial colonisation and immune development proceed in parallel and are interdependent. Commensal organisms educate the neonatal immune system, inducing regulatory T-cell populations and establishing the immune tolerance that prevents inflammatory and autoimmune disorders; infant-type bifidobacteria appear central to this education, acting in part through short-chain fatty acid metabolites [37,41].

**Adipocyte development.** Adipose tissue is laid down in this window, and its cellularity and distribution are programmed by early nutritional conditions. Animal work demonstrates that maternal dietary manipulation alters adipogenic transcription factors such as PPARγ and C/EBPα in offspring, and can produce either increased adipocyte number or adipocyte hypertrophy depending on the timing and severity of the exposure [42]. The reasons for these divergent phenotypes remain incompletely understood, and how far the findings extend to human infants is unresolved.

Breastfeeding as a protective link in the chain. Breastfeeding sits at the intersection of all three processes, and its relation to later obesity has been extensively studied. A meta-analysis of 25 studies comprising 226,508 participants across 12 countries reported that breastfeeding was associated with reduced odds of childhood obesity (adjusted OR 0.78, 95% CI 0.74–0.81), with a stepwise gradient of decreasing risk across increasing duration [43]. A subsequent meta-analysis of 26 publications and 332,297 participants found pooled odds of 0.83 for ever-breastfed preschoolers, with a dose–response relationship in which each additional month of breastfeeding was associated with a 4% reduction in risk, and with exclusive breastfeeding conferring the largest benefit (OR 0.53) [44]. Notably, the protective effect became apparent only after approximately six months of sustained feeding [44]. Candidate mechanisms include the bioactive constituents of human milk (leptin, adiponectin, ghrelin, insulin-like growth factor-1), together with the microbiome and behavioural pathways described above [45]. The association is not undisputed; some recent systematic appraisals conclude that the evidence for a duration effect remains insufficient, and residual confounding by maternal characteristics is difficult to exclude. Formula, though classified as ultra-processed, cannot be equated with discretionary ultra-processed products; conflating the two is precisely the error that Section 2.1 warned against.

### 3.3. Complementary Feeding

The introduction of solid foods opens the third window, and it is arguably the point at which discretionary ultra-processed exposure first becomes substantial.

**Taste learning.** Not all taste responses are learned. The basic tastes carry a strong innate component: sweet, umami, and salty stimuli are innately preferred, while bitter and many sour stimuli are innately rejected, a bias plausibly shaped by the evolutionary value of energy and minerals and the toxicity signalled by bitterness [46]. Children prefer higher levels of sweet and salt than adults do, and these preferences decline toward adult levels only in adolescence [46]. Onto this innate template, experience writes: flavour preferences are also learned, and the learning begins before the first solid meal. In a classic experiment, infants whose mothers consumed carrot juice during late pregnancy or early lactation showed greater acceptance of carrot-flavoured cereal at weaning than unexposed infants, demonstrating that flavours transmitted through amniotic fluid and breast milk are acquired [47]; a systematic review has since consolidated the evidence for this prenatal and early-postnatal flavour bridge [48]. The infant arrives at weaning already primed.

**Food preferences.** What is offered during weaning consolidates into what is preferred thereafter. Commercial complementary foods expose the infant to ultra-processed formulations during exactly the window in which preferences are being established [19,26], and nutritional and promotional analyses of these products confirm both their ultra-processed character and the marketing claims that accompany them [49]. Repeated early exposure to intensely sweet or salty formulations plausibly raises the hedonic set-point for these tastes. The longitudinal human data supporting this durable-conditioning hypothesis remain partial, and it should be presented as plausible rather than demonstrated.

**Eating behaviour.** Beyond preference, the weaning period shapes the regulation of intake itself. Appetitive traits (responsiveness to food cues, satiety responsiveness, eating rate) are measurable in early childhood and predict subsequent adiposity, and ultra-processed consumption has been prospectively associated with these traits [50]. Weaning, often treated as nutritionally forgiving on the grounds that the child will “grow out of it”, may instead be a high-leverage window in which both what is preferred and how it is eaten are set.

## 4. Biological Mechanisms

**Figure 2:**
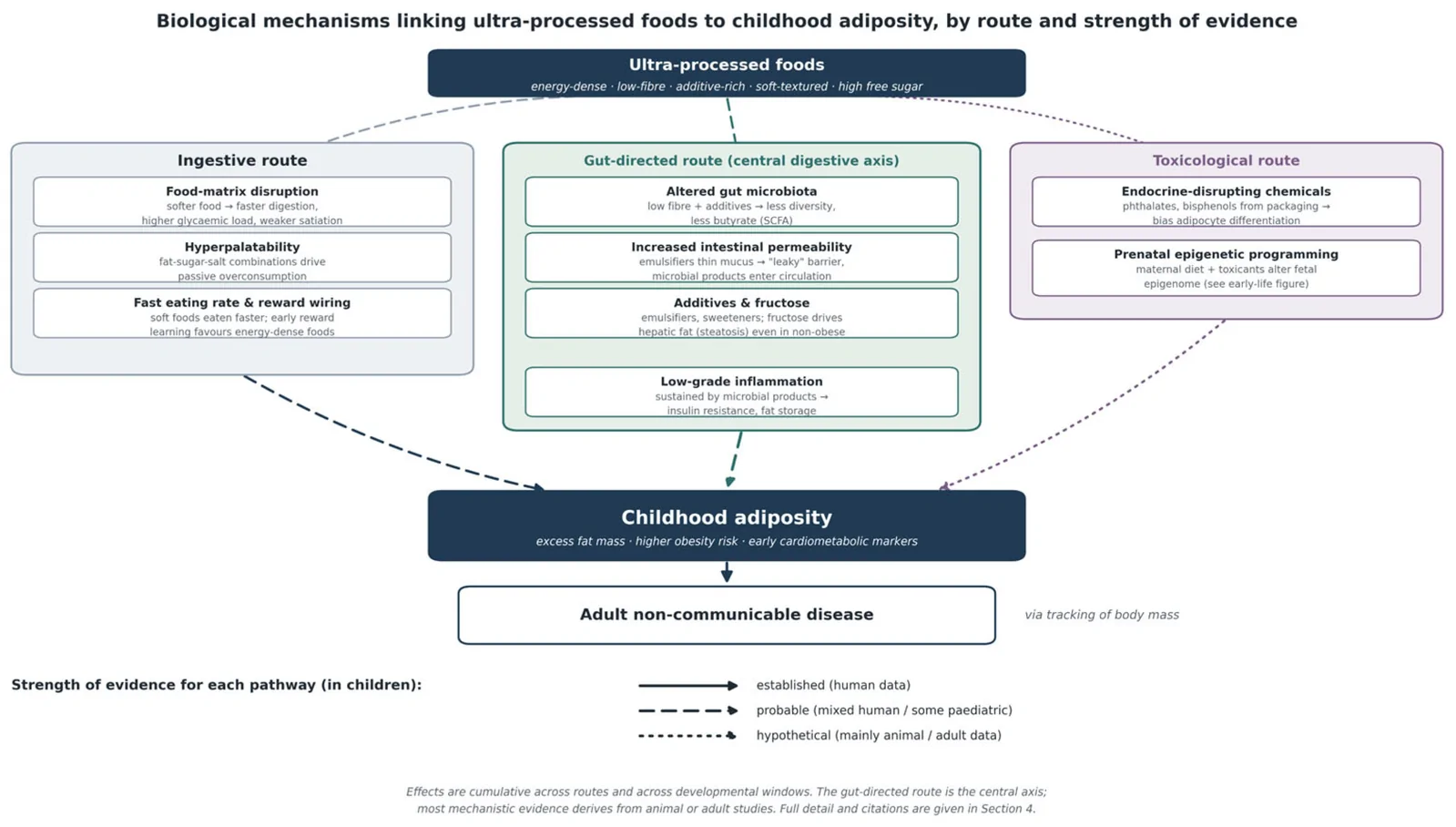
Biological mechanisms linking ultra-processed foods to childhood adiposity. The eight mechanisms are grouped by route—ingestive, gut-directed, and toxicological—and each is shown with its principal effect. The gut-directed route forms the central digestive axis. Line style indicates the strength of the evidence for each pathway (established, probable, or hypothetical); effects are cumulative across routes and windows, and most mechanistic evidence derives from adult or animal studies. Full detail and citations are given in Section 4.

If ultra-processed foods influenced body weight solely by being energy-dense and palatable, they would warrant no separate mechanistic account; the established physiology of positive energy balance would suffice. The case for treating them as a distinct exposure rests on evidence that several of their attributes act through routes at least partly independent of energy content. Eight such routes are considered below. They are not independent of one another, and it is worth stating the connections at the outset rather than allowing the reader to infer eight parallel pathways where there are in fact a smaller number of interlocking ones. Matrix disruption operates in substantial part by accelerating eating rate Additives act on the gut microbiota and, through it and directly, on intestinal permeability Hyperpalatability describes a property of the food; the neurobiology of reward describes what that property does to the brain. Endocrine-disrupting chemicals connect back to the epigenetic programming of Section 3.1. Two of these routes are, however, more central than the rest. The assembly of the gut microbiota and the integrity of the intestinal barrier form the digestive axis through which much of the adiposity signal is plausibly transmitted: additives, displaced fibre and altered eating behaviour converge on the gut, and the low-grade inflammation and disrupted appetite signalling that follow are the proximate links to excess fat mass. The remaining routes are best read as feeding into, or modulating, this digestive core. What follows is therefore a set of vantage points on an integrated process, organised around the gut, not a catalogue of parallel pathways. Table 1 sets out the eight mechanisms against the convergent processes depicted in Figure 2, together with the strongest supporting evidence for each and whether that evidence is paediatric, adult or animal in origin.

A second qualification applies throughout. The great majority of the experimental evidence has been generated in adults or in animal models. Where paediatric data exist, we say so; where they do not, the mechanism is an extrapolation, and its plausibility should not be mistaken for demonstration. This asymmetry is the empirical counterpart of the developmental argument in Section 3: the window in which these mechanisms would matter most is precisely the window in which they have been least studied.

### 4.1. Food Matrix Disruption

The physical structure in which nutrients are embedded, the food matrix, shapes the rate and site of digestion, the glycaemic response, and the satiation that ends a meal. Ultra-processing systematically degrades this structure. Milling, extrusion, and homogenisation disrupt intact cell walls and fibre architecture, increasing the accessibility of starch to digestive enzymes and accelerating glucose release. The resulting product is softer, and softness reduces the oral processing a food demands.

The most direct evidence comes from the inpatient controlled-feeding trial of Hall and colleagues, in which 20 weight-stable adults received ultra-processed and unprocessed diets for two weeks each in randomised crossover order, the meals matched for available energy, energy density, macronutrients, sugar, sodium, and fibre [51]. On the ultra-processed arm, participants spontaneously consumed roughly 500 kcal more per day and gained approximately 0.9 kg, whereas they lost a comparable amount on the unprocessed arm [51]. Because the matching design isolates attributes of processing from nutrient composition, the surplus intake is mechanistically informative. A more recent randomised crossover trial, in which both diets adhered to healthy dietary guidelines but differed in processing, found greater weight loss on the minimally processed arm, indicating that processing retains an effect even when nutrient quality is held to recommended standards [52].

The paediatric relevance is inferential rather than demonstrated. Children’s satiety responsiveness and capacity for intake self-regulation differ from those of adults, and the weaning diet is by design a progression of textures. No trial of comparable rigour has been conducted in children, and the magnitude of the matrix effect in a two-year-old is unknown. The pathway to adiposity is nonetheless coherent: a softer matrix that is eaten faster and digested more rapidly favours a larger energy intake per meal and a higher postprandial glycaemic load, both of which promote fat deposition over time.

### 4.2. Hyperpalatability

Ultra-processed foods are frequently engineered to combinations of fat, sugar, and salt that are rarely found together in whole foods and that appear especially effective at driving consumption beyond homeostatic need. Palatability is not a defect of these products but their design specification. In infancy and early childhood, the point acquires additional force, because the hedonic set-point for sweetness and saltiness is not fixed but learned, as Section 3.3 described. A food that is hyperpalatable to an adult acts on a preference structure already formed; the same food offered at eighteen months acts on one still forming.

The evidence that hyperpalatable formulations can override satiety signalling and promote intake in the absence of energy need is reasonably secure. The stronger claim, that such foods meet criteria for addictive substances, is contested and is considered in Section 4.8. Either way, the route to obesity runs through passive overconsumption: palatability that detaches intake from need adds energy that is stored as fat.

### 4.3. Gut Microbiota

The gut microbiota links microbial activity to the central control of intake, mediating several of the routes considered here, and it does so most consequentially during the assembly described in Section 3.2. Bacterial fermentation of non-digestible carbohydrate yields short-chain fatty acids, which act on enteroendocrine cells to modulate the secretion of appetite-regulating hormones (glucagon-like peptide-1, peptide YY, and ghrelin among them) and thereby influence energy absorption, satiety, and adiposity [53]. These metabolites reach central circuits through enteroendocrine, vagal, and humoral routes, forming a bidirectional microbiota–gut–brain axis [54].

A dietary pattern high in ultra-processed foods reshapes this ecosystem in at least two ways. It displaces fermentable fibre, reducing short-chain fatty acid production—most consequentially butyrate, the principal fuel of colonocytes and a signal that supports epithelial barrier integrity and restrains mucosal inflammation, so that its depletion links the ultra-processed diet to the barrier failure described in Section 4.4; and it introduces additives capable of altering microbial composition directly, as Section 4.5 sets out. In a murine model, the emulsifiers carboxymethylcellulose and polysorbate 80, administered at concentrations modelling human dietary exposure, altered microbial composition, promoted bacterial encroachment on the epithelium, and induced low-grade intestinal inflammation with increased adiposity and dysglycaemia [55]. An ex vivo screen of 20 commonly used emulsifiers found that many, not only the two best-studied, detrimentally reshaped human microbial communities [56], and a controlled-feeding study in humans reported that carboxymethylcellulose perturbed the gut microbiota and the faecal metabolome [57].

The mechanism is biologically plausible and supported by animal and adult human data. Its operation in children specifically remains inferential, and the direction and magnitude of any effect from ultra-processed exposure in early life have not been established.

### 4.4. Intestinal Permeability

The intestinal epithelium regulates the passage of luminal contents into the circulation through intercellular tight junctions composed of occludin, claudins, and zonula occludens proteins. Zonulin is the principal known physiological modulator of these junctions, and its upregulation leads to an uncontrolled influx of dietary and microbial antigens across the epithelium [58]. When bacterial products, chiefly lipopolysaccharide, translocate into the portal circulation, they engage the LPS/CD14 system on adipocytes and hepatocytes and initiate a low-grade inflammatory state. In the experiments that established this pathway, four weeks of high-fat feeding in mice raised plasma lipopolysaccharide two- to threefold above baseline, a threshold the authors termed metabolic endotoxaemia, and continuous subcutaneous infusion of lipopolysaccharide alone was sufficient to induce weight gain, insulin resistance, and hepatic steatosis [59]. The inflammatory tone so established dysregulates insulin signalling and, in animal models, hypothalamic leptin signalling, contributing both to insulin resistance and to the dysregulation of appetite.

Two observations connect this pathway to ultra-processed exposure in the developing child. The first is that emulsifiers thin the protective mucus layer that separates bacteria from the epithelium, and mucus thinning precedes bacterial encroachment and inflammation in the murine model described above [55]. The second is developmental and comes from human infants: in a birth cohort of 349 children, faecal extracts from infants whose microbiome had matured precociously promoted enterocyte barrier dysfunction in vitro, and these infants carried a higher relative risk of overweight or obesity at two years [60].

We enter a caveat here that applies with particular force. Much of the barrier literature in obesity is cross-sectional and adult, biomarkers of permeability such as serum zonulin are of contested specificity, and the direction of causation between barrier dysfunction, dysbiosis, and adiposity is not settled. That increased permeability accompanies obesity is well documented; that it precedes and causes it in children is not.

### 4.5. Food Additives

Non-nutritive additives define NOVA group 4 and constitute, in commercial infant and toddler foods, the dominant reason for ultra-processed classification [19]. This is not a peripheral observation. It means that the exposure of greatest concern in the first two years of life is, in large measure, an exposure to emulsifiers, thickeners, and flavour enhancers rather than to sugar or fat.

The emulsifier evidence has been set out in Section 4.3 and Section 4.4 and need not be repeated; it constitutes the strongest mechanistic case for an additive effect independent of nutrient composition [55,56,57]. Reviews of dietary emulsifier exposure note that intake is widespread and difficult for consumers to gauge [61]. Non-nutritive sweeteners have been reported to influence glucose tolerance through microbiota-dependent routes, although the human evidence is inconsistent. Their use in children raises distinct concerns: they are increasingly present across the food and beverage supply, including products marketed to the young, yet the long-term paediatric evidence remains thin and contested [62]. There is reasonable agreement that non-sugar sweeteners are inadvisable before three years of age and best avoided up to five, but beyond that the balance of benefit and risk is unresolved, and the World Health Organization’s 2023 guidance advising against their use for weight control has itself been subject to critical appraisal [62,63].

The paediatric salience is twofold: the gut microbiome is assembling, so perturbations may carry more durable consequences than in a mature ecosystem; and additive intake per kilogram of body weight is plausibly higher in young children. Direct paediatric data on additive exposure and metabolic outcome are, at present, absent. This is among the clearest research gaps identified by this review.

### 4.6. Endocrine-Disrupting Chemicals

The fourth mechanistic strand concerns substances that are not deliberate ingredients: contaminants generated during processing and chemicals migrating from packaging. Thermal processing of starch-rich foods generates acrylamide, and in a nationally representative United States sample, higher dietary energy from ultra-processed foods was associated with higher haemoglobin biomarkers of acrylamide exposure [6]. Of particular interest for adiposity are the obesogens, a concept introduced by Grün and Blumberg to describe endocrine-disrupting chemicals that promote weight gain by altering adipogenesis, lipid homeostasis, and the neuroendocrine control of appetite [64]. Mechanistic, animal, and epidemiological evidence implicates plasticisers such as certain phthalates and bisphenols in adipogenic and metabolic dysregulation, with prenatal and early-life exposure of particular concern, although the authors of such syntheses are careful to flag persisting controversies [65].

Ultra-processed foods are a plausible quantitative source of these exposures: extended industrial contact and packaging favour chemical migration, and in NHANES data, higher ultra-processed intake was associated with higher urinary concentrations of phthalate metabolites, an association the authors noted was especially relevant for children, who are both more vulnerable to such toxicants and tend to consume more ultra-processed food than adults [66]. In a cohort of pregnant women, ultra-processed and fast-food intake was associated with phthalate exposure and statistically mediated part of the socioeconomic disparity in that exposure [67].

This pathway returns us to Section 3.1. Endocrine-disrupting chemicals act, in substantial part, by altering nuclear receptor signalling and DNA methylation at metabolic loci, the same epigenetic machinery through which maternal nutrition programmes the fetal phenotype [25]. The nutritional and toxicological routes of early-life programming are not separate; they converge on the epigenome. That convergence is, in our view, the most interesting and least explored aspect of ultra-processed exposure in the prenatal window.

The evidence here remains associational, confounding is severe, and causal attribution is premature. It is the least mature of the eight mechanisms and should be read as such. Where an effect exists, its proposed route to adiposity is hormonal rather than caloric: endocrine disruptors are thought to bias adipocyte differentiation and lipid storage independently of energy intake.

### 4.7. Eating Rate

Eating rate deserves separate treatment because the evidence for it is unusually good and because its implications are uncomfortable for a simple account of ultra-processing.

Softer texture raises eating rate, and faster eating raises energy intake before satiation intervenes. Teo and colleagues showed that texture-based differences in eating rate influenced energy intake for both minimally processed and ultra-processed meals, with the effect falling more on within-meal satiation than on later satiety [68]. More directly, in a two-week randomised crossover trial in which both arms were ultra-processed but differed only in texture-derived eating rate, the slower-eating-rate diet reduced daily intake by several hundred kilocalories, an effect consistent across participants and sustained across the fortnight [69].

The implication should be stated plainly rather than buried. If a slow-textured ultra-processed diet and a fast-textured one diverge sharply in energy intake, then a meaningful part of what is attributed to ultra-processing may in fact be texture and eating rate, properties that are common in, but not unique to, ultra-processed foods, since the interrelations between food form, texture, and matrix govern both intake and the metabolic response to a meal [70]. The honest framing is not “beyond energy density” alone but “beyond energy density, and in part beyond processing itself”. This does not dissolve concern about ultra-processed foods, which reliably cluster the very textures that drive fast eating; it relocates part of the mechanism and should temper deterministic claims.

For paediatrics the corollary is practical. Texture is one of the few features of the infant diet that clinicians and parents directly control, and the progression from purée to solids is already a routine part of anticipatory guidance. If eating rate carries a substantial share of the effect, then advice about texture may be more actionable than advice about NOVA category. The mechanism reaches obesity directly: a faster eating rate weakens the satiation signal that would otherwise end a meal, so that more energy is consumed before satiety registers.

### 4.8. Neurobiology of Reward

Highly palatable foods recruit orosensory and post-ingestive dopaminergic circuits that influence the desire to eat [71]. Some authors argue that ultra-processed foods meet established criteria for addictive substances, pointing to dopaminergic signalling, craving, and withdrawal-like phenomena, and have proposed clinical and policy responses on that basis [72,73]. A systematic review and meta-analysis using the Yale Food Addiction Scale estimated that approximately 15% of children and adolescents meet criteria for food addiction [74], one of the few strands of this mechanism supported by direct paediatric data. This estimate warrants caution: the Yale Food Addiction Scale was developed and validated in adults, and its psychometric performance and construct validity in children and adolescents remain debated, so paediatric prevalence figures derived from it should be read as provisional.

The construct is contested, and the evidence for its central premise is mixed. When Darcey and colleagues measured striatal dopamine responses to an ultra-processed milkshake using positron emission tomography, the responses were highly variable, were not significantly related to adiposity, and were substantially smaller than those elicited by addictive drugs, being undetectable in many participants [75]. The honest reading is that hyperpalatable formulations can engage reward circuitry and override satiety signalling, but that the stronger addiction framing remains a hypothesis rather than a settled mechanism.

The developmental dimension supplies what the addiction debate lacks. Whether or not ultra-processed foods are addictive in the technical sense, they are demonstrably capable of shaping the reward valuation of food during the period when that valuation is being established [47,48]. A mechanism that acts by conditioning preferences early does not require the addiction framework to be consequential. Its bearing on adiposity is indirect but durable: reward learning that favours energy-dense ultra-processed foods in early life shapes the dietary pattern that carries obesity risk forward.

### 4.9. Fructose and Hepatic Metabolism

A ninth route deserves separate treatment because its principal target is not adipose tissue directly but the liver, and because the sugar that drives it is added to precisely the products children consume most. High-fructose corn syrup and other free-fructose sources are ubiquitous in sweetened beverages, breakfast cereals, flavoured dairy and confectionery, and paediatric fructose intake has risen in parallel with these products. Unlike glucose, fructose is metabolised almost entirely in the liver, where it bypasses the rate-limiting steps of glycolysis and provides an unregulated substrate for de novo lipogenesis; the result is hepatic fat accumulation that can occur, notably, in children who are not obese [76].

The relevance to the present argument is threefold. First, fructose is a plausible mechanistic link between ultra-processed intake and the rising paediatric burden of metabolic dysfunction-associated steatotic liver disease, a condition once considered adult but now documented in normal-weight children exposed to high free-sugar diets [76,77]. Second, the pathway is not confined to the liver: excess fructose alters the gut microbiota and increases intestinal permeability, allowing microbial products to reach the portal circulation and sustain the low-grade inflammation described in Section 4.4, so that the hepatic and the digestive-axis mechanisms are continuous rather than separate. Third, fructose acts on adipose tissue physiology and on uric acid metabolism in ways that favour fat storage and insulin resistance [77]. As with the other mechanisms, the human paediatric evidence is still largely observational and much of the mechanistic detail derives from animal and adult work; but the convergence of a rising exposure, a defined hepatic pathway, and a paediatric disease of growing incidence makes fructose one of the more clinically consequential components of the ultra-processed diet.

## 5. Epidemiological Evidence in Children

The mechanistic and developmental arguments would carry little weight without an epidemiological signal. That signal is present, but its strength varies systematically with study design, and the variation is itself informative. We therefore consider the evidence by design rather than by outcome, moving from the weakest inferential footing to the strongest. Table 2 summarises representative studies and their effect estimates.

### 5.1. Cross-Sectional Studies

Cross-sectional surveys are the most numerous and the least persuasive. They establish that ultra-processed intake and adiposity co-occur, which nobody disputes, and they are structurally unable to distinguish the hypothesis that ultra-processed foods promote adiposity from the hypothesis that children with adiposity eat, or report eating, differently. In United States adolescents, the highest compared with the lowest quartile of ultra-processed consumption carried between 45% and 63% higher odds of total, abdominal, and visceral adiposity [78]. In the China Health and Nutrition Survey, sustained ultra-processed intake at the highest exposure level was associated with roughly twofold higher odds of overweight or obesity [79].

Such estimates are consistent in direction across settings, which lends a degree of robustness through replication. They are also precisely the estimates most vulnerable to reverse causation and to differential misreporting, as Section 6 sets out. A systematic review of paediatric studies found that cross-sectional analyses generally did not detect the associations that longitudinal analyses did [80], a pattern that, read charitably, indicates that a single snapshot fails to capture a cumulative exposure, and read sceptically, indicates that the association appears when investigators have more analytical degrees of freedom. Either way, the cross-sectional tier is best read as establishing co-occurrence rather than direction, which is why the weight of the causal argument falls on the cohort evidence that follows.

### 5.2. Cohort Studies

Prospective cohorts carry the burden of the causal argument in this literature. In the Avon Longitudinal Study of Parents and Children, a British birth cohort, higher childhood ultra-processed consumption was associated with steeper trajectories of adiposity through to early adulthood [81]. A Brazilian cohort linked ultra-processed intake to fat-mass index between ages six and eleven [82], and a prospective Portuguese study related ultra-processed consumption to appetitive traits and body-mass index in children [50]. In the German KiGGS cohort, each 10% increment in ultra-processed intake was associated with approximately 12% higher odds of overweight, with the highest quartile carrying about 49% higher odds than the lowest [83].

Two features of this literature deserve emphasis. The first is that outcomes do not move together: in a prospective cohort of Chilean preschoolers, ultra-processed intake was associated with adiposity but not with metabolic indicators [84]. The adiposity phenotype is not monolithic, and an exposure that shifts fat mass need not shift glycaemia or lipids on the same timescale. The second is the maternal cohort evidence discussed in Section 3.1, in which ultra-processed consumption during the postnatal period predicted offspring overweight, while consumption during the peripregnancy window did not [35]. Prospective design does not by itself resolve the confounding by shared environment that this dissociation exposes.

### 5.3. Meta-Analyses and Pooled Estimates

Pooled estimates place the magnitude of association within a broad range. A meta-analysis of observational studies across age groups reported a pooled effect for obesity of 1.26 (95% CI 1.13–1.41) and a smaller but significant effect for overweight, drawing predominantly on cross-sectional data [85]. A meta-analysis restricted to adolescents, pooling 23 studies across five continents, found higher ultra-processed consumption associated with greater odds of overweight or obesity, with a pooled odds ratio of 1.63 (95% CI 1.36–1.95), and associations exceeding a twofold increase in the most recent studies [86]. Umbrella syntheses across all ages report broadly convergent associations with adverse metabolic outcomes, though effect sizes attenuate under stricter adjustment [87].

The spread between an odds ratio of 1.26 and one of 2.09 is not a detail. It reflects genuine heterogeneity in exposure definition, adiposity measurement, population, and covariate adjustment, and it should discipline the confidence with which any single figure is quoted. A systematic review focused on children and adolescents concluded that the association is more robust in adults than in children [88], and this adult–child gradient is the empirical counterpart of the mechanistic asymmetry noted throughout Section 4. Louie has argued, provocatively but not unreasonably, that the methodological limitations of the underlying observational studies are severe enough that their systematic pooling should be suspended pending improvement [89].

### 5.4. Intervention Studies

Randomised trials are what the field most conspicuously lacks. In adults, the inpatient controlled-feeding paradigm has produced the strongest evidence of a causal effect of processing [51,52]. In children, the paediatric literature contains few comparable trials. One open-label randomised study in children and adolescents found that adding ultra-processed avoidance to a guideline-based diet improved weight and metabolic measures, but the open-label design, modest sample, and absence of blinding constrain causal inference [90]. A second small trial reinforces both the signal and its limits: a school-based cluster intervention that reduced the availability of ultra-processed snacks and beverages achieved modest reductions in their consumption, but measured environmental change rather than an isolated food effect [91].

The reasons for this scarcity are practical and ethical rather than conceptual. Controlled feeding over developmentally meaningful durations is difficult to justify and harder to execute in children, and cluster-randomised environmental interventions (in nurseries, schools, or supplemental feeding programmes) measure the effect of a policy rather than of a food. Until such trials exist, the paediatric evidence base rests on observational data whose limitations we now consider directly. The scarcity is therefore structural rather than incidental, and it defines the single clearest priority for future paediatric research: designs that can approximate causal inference without the ethical and practical barriers of prolonged controlled feeding in children.

**Table 2:** Selected studies of ultra-processed food (UPF) intake and adiposity in children and adolescents, by design. RR, relative risk; OR, odds ratio; NS, not significant; Q, quartile. Evidence tiers indicate the weight that each design can carry and are assigned from the design alone: Tier 1, prospective cohort or randomised trial, in which exposure precedes outcome; Tier 2, quantitative or narrative synthesis of observational studies, in which the inherited limitations of the pooled designs apply; Tier 3, cross-sectional survey, in which temporality cannot be established and reverse causation remains possible. The tiers are not a formal risk-of-bias appraisal: no Newcastle–Ottawa, ROBINS-I or RoB 2 assessment was undertaken, such appraisal falling outside the scope of a narrative review. Rows within a tier are not interchangeable, and the study-specific limitations are given in the final column.

Study	Design; Population	Evidence Tier	Outcome	Effect Estimate	Main Limitation
Wang et al. 2022 [35]	3 prospective cohorts; 19,958 mother–child pairs (US)	Tier 1—prospective cohort	Offspring overweight/obesity	RR 1.26 (1.08–1.47), highest vs. lowest maternal UPF (postnatal); NS for peripregnancy	Observational; residual confounding by shared postnatal environment
Chang et al. 2021 [81]	Birth cohort (ALSPAC), UK, to age 24	Tier 1—prospective cohort	Adiposity trajectory	Steeper adiposity gain with higher childhood UPF intake	Single cohort; self-reported diet; attrition to age 24
Costa et al. 2021 [82]	Prospective cohort; children 6–11 y (Brazil)	Tier 1—prospective cohort	Fat-mass index	Positive association with UPF intake	Single cohort; cross-sectional diet measure
Zancheta Ricardo et al. 2024 [84]	Prospective cohort; preschoolers (Chile)	Tier 1—prospective cohort	Adiposity; metabolic markers	Associated with adiposity; not with metabolic markers	Single cohort; short follow-up; residual confounding
Figueiredo Barata et al. 2025 [83]	Longitudinal cohort (KiGGS), Germany	Tier 1—prospective cohort	Overweight/obesity	OR ≈ 1.12 per 10% UPF; Q4 vs. Q1 OR ≈ 1.49	Observational; residual confounding; misclassification
Liu et al. 2022 [78]	Cross-sectional (NHANES); US adolescents	Tier 3—cross-sectional	Total/abdominal/visceral adiposity	OR 1.45–1.63, highest vs. lowest quartile	Cross-sectional; reverse causation cannot be excluded
Li & Shi 2025 [79]	China Health and Nutrition Survey	Tier 3—cross-sectional	Overweight/obesity	≈2-fold higher odds at highest exposure	Cross-sectional; self-reported intake
Askari et al. 2020 [85]	Meta-analysis; observational, all ages	Tier 2—synthesis of observational data	Overweight; obesity	Pooled OR 1.02 (overweight); 1.26 (1.13–1.41) (obesity)	Pooled observational; heterogeneity; NOVA misclassification
Aweke et al. 2026 [86]	Meta-analysis; 23 studies, five continents	Tier 2—synthesis of observational data	Overweight/obesity	Pooled OR 1.63 (1.36–1.95); >2 in recent studies	Pooled observational; between-study heterogeneity
De Amicis et al. 2022 [80]	Systematic review; children/adolescents	Tier 2—synthesis of observational data	Adiposity	Positive in longitudinal (>4 y); null in cross-sectional	Design-dependent (null cross-sectional); few long cohorts
Chen et al. 2022 [90]	Open-label RCT; children/adolescents	Tier 1—randomised, unblinded	Weight; metabolic measures	Improvement with UPF avoidance added to guideline diet	Open-label; small sample; no blinding

## 6. Strengths and Limitations of Current Evidence

The strengths of the literature are real and should be stated before its weaknesses. The association between ultra-processed intake and adiposity has been replicated across continents, income levels, and dietary cultures; it survives adjustment for the obvious confounders in most analyses; it is graded by exposure in several cohorts; and it is accompanied by a set of biologically plausible mechanisms, at least two of which have experimental support in humans. This is a more coherent evidence package than exists for most single dietary exposures. Six limitations nonetheless constrain what may be concluded from it.

NOVA misclassification. The exposure is defined by processing history rather than by any calibrated dose of a harmful constituent. Inter-rater agreement in assigning foods to NOVA groups is poor, with Fleiss’ kappa around 0.32 [15]. The category aggregates products of very different likely effect [16], and in paediatric research it aggregates infant formula with confectionery, as Section 2.1 argued at length [18,19,20,21,22,23,24,25]. Even a true causal effect of certain ultra-processed foods would be diluted, and potentially distorted, by the inclusion of others.

Residual confounding. Ultra-processed intake is strongly socially patterned, tracking with household income, parental education, food insecurity, and the local food environment, each of which independently predicts childhood adiposity. The patterning extends to correlated exposures: maternal ultra-processed intake mediates part of the socioeconomic gradient in phthalate exposure [67], and the higher-obesity-risk infant microbiome described earlier clustered with lower socioeconomic position [60]. Beyond social position, childhood adiposity is multifactorial, and ultra-processed intake does not act in isolation: physical inactivity, short or disrupted sleep, and screen time all track with higher ultra-processed consumption and independently predict weight gain. Few paediatric studies simultaneously measure and adjust for all of these potential confounders.

**Reverse causation.** Cross-sectional associations are vulnerable to the possibility that children with higher adiposity alter their intake, or their reporting of it. The concern applies with particular force to the design that supplies most of the paediatric estimates.

Dietary assessment. Intake is captured almost universally through recall or food-frequency instruments, dependent on parental report for younger children and adolescent self-report thereafter. Misreporting of energy intake is pervasive in this age range, with roughly half of individuals misclassified in some validation studies, and the bias is patterned by body weight: children and adolescents with higher adiposity tend to under-report, and to do so selectively for energy-dense items of precisely the kind that dominate the ultra-processed category [92,93]. Misreporting attenuates diet–obesity associations unless explicitly modelled [94]. Self-report instruments yield, at best, valid intake estimates at the group rather than the individual level. Validated biomarkers of ultra-processed exposure remain investigational.

**Lack of randomised controlled trials.** As Section 5.4 set out, the paediatric trial evidence is minimal, and the adult trials that do exist cannot be assumed to transfer to a developing organism whose intake regulation differs.

Mechanistic evidence mainly from animals. The mechanisms in Section 4 rest substantially on murine models and ex vivo systems. The emulsifier work, the endotoxaemia work, and the adipocyte programming work are all, in their decisive form, animal science. Direct paediatric mechanistic demonstration is confined to a handful of strands (the early learning of flavour, the food-addiction prevalence estimate, the infant microbiome cohort), and even these are associational.

These limitations do not nullify the evidence; they calibrate it. What the literature supports is a coherent case, not a closed one, and the distinction is worth preserving.

## 7. Implications for Paediatric Practice

The heterogeneity of the ultra-processed category counsels specificity over slogans, since blanket advice to avoid ultra-processed foods is hard for families to act on and risks stigmatising items that are necessary or benign.

Counselling parents. Advice is most likely to be actionable when it names products rather than categories. The discretionary items most consistently implicated (sugar-sweetened beverages, confectionery, energy-dense sweet and savoury snacks) can be identified by a parent without reference to a classification system. Advice is also most likely to be effective when it is delivered early, because preferences and dietary patterns form in the windows described in Section 3 and track forward [81]. Counselling during pregnancy, infancy, and the preschool years is likely to yield more than the same effort directed at an older child whose patterns have consolidated. Guidance should acknowledge the socioeconomic gradient of the exposure: recommendations that require time, money, or cooking facilities that a family does not have will widen rather than narrow inequalities.

Breastfeeding should be supported for the reasons set out in Section 3.2, where the dose–response relationship with reduced obesity risk is among the better-quantified findings in this review [43,44]. Where breastfeeding is not possible, formula is the appropriate and necessary alternative, and clinicians should say so plainly; its NOVA classification is a taxonomic artefact rather than a clinical finding (Section 2.1), and discouraging formula use on that basis would harm the infants who depend on it.

Complementary feeding. Weaning is a high-leverage window rather than a nutritionally forgiving one. Home-prepared foods of varied texture are preferable to commercial pouches and infant snacks where circumstances allow, both because commercial products carry higher sugar and sodium and because their additive burden drives their ultra-processed classification [19]. Texture deserves explicit attention: progression toward foods requiring chewing may attenuate the rapid-eating phenotype described in Section 4.7. Repeated exposure to bitter and sour tastes, and delay of intense sweetness, are consistent with what is known about early flavour learning [47,48].

Beverage recommendations. Sugar-sweetened beverages are the component of the ultra-processed category with the most mature causal literature and the clearest policy evidence, and they should be the first target of both counselling and regulation. Energy drinks warrant specific attention in adolescence given their cardiovascular and neuropsychiatric adverse-event profile in the young [95]. Water and unsweetened milk remain the appropriate beverages for children; the growing category of toddler and growing-up formulas falls outside the WHO marketing code and is not nutritionally necessary [18].

Label reading. Front-of-pack claims on children’s products are capable of misleading caregivers, and health-oriented claims appear disproportionately on products of poor nutritional quality [96]. Practical guidance (read the ingredient list rather than the front of the pack, treat a long list of unfamiliar names as a signal, distrust claims of nutritional superiority on products marketed to children) is more useful than an instruction to avoid a NOVA category the parent cannot operationalise.

**Food quality.** Finally, clinicians should be honest about the limits of the evidence: how much of the effect attributed to ultra-processing reflects texture, additives, displacement of whole foods, or the social circumstances that predict both remains unresolved. Advice grounded in whole-food dietary patterns, adequate fibre, and family meals is defensible on grounds that do not depend on that question.

## 8. Future Directions

Individual counselling is necessary but insufficient against an exposure whose drivers are structural, and several lines of research and policy warrant attention.

Structural levers deserve priority in future work, since the exposure’s drivers lie beyond individual choice. Several are already partly evidenced and now require paediatric-specific evaluation: mandatory reformulation, which lowers population intakes more reliably than voluntary schemes but whose removal of emulsifiers from infant products remains untested [97,98,99]; fiscal and labelling measures, whose combined deployment in settings such as Chile was followed by falling purchases of sugar-sweetened beverages and for which coordinated policy roadmaps now exist [100,101,102]; marketing restriction, including closure of the gap in the WHO code that leaves growing-up formulas and packaged baby foods uncovered [18,103,104]; and the extension of front-of-pack warnings and additive declaration to infant and toddler products, addressing the concern raised in Section 4.5. International surveillance underlines the urgency [105,106]. School food environments, reaching children while patterns consolidate, offer a natural setting for the cluster-randomised trials the field lacks.

The first 1000 days. The most important research direction follows from the argument of this review. If ultra-processed exposure acts principally through developmental programming, then the decisive studies are birth cohorts with prospectively measured maternal and infant ultra-processed intake, disaggregated by product type, linked to microbiome sampling, epigenetic profiling, and body-composition assessment through childhood. Such cohorts would allow the chain proposed here (prenatal nutrition, breastfeeding, complementary feeding, ultra-processed exposure, microbiota, inflammation, immune maturation, epigenetic change, adiposity, adult disease) to be tested rather than assumed. Several existing cohorts hold the biological samples required. What is presently lacking is exposure measurement adequate to the question.

### Healthy Eating and Lifestyle

A review preoccupied with what children should avoid risks neglecting what they should eat, yet the two are inseparable: the displacement of whole foods is one of the ways in which an ultra-processed diet exerts harm. The dietary pattern associated with lower adiposity in childhood is well characterised and consistent across evidence syntheses. It is built on vegetables and fruit, legumes, whole grains, fish that is not fried, eggs, dairy—fermented products such as yogurt in particular—poultry that is not fried, and water as the default drink, with red and processed meats, sugar-sweetened beverages and energy-dense snacks kept low [107,108].

Two features of this pattern deserve emphasis in the paediatric context. First, several of its components overlap with foods that are technically ultra-processed yet nutritionally valuable, as Section 3 argued: wholegrain breakfast cereals, wholegrain packaged bread and commercial plain yogurt can all belong to a healthy diet, which is why advice framed around whole-food quality serves children better than advice framed around the NOVA label alone. Second, diet does not act in isolation. The consensus paediatric recommendations that accompany these dietary targets place regular physical activity, adequate sleep and limited sedentary time alongside food choice, on the reasonable grounds that energy balance and cardiometabolic health in childhood are shaped by the whole pattern of daily life rather than by any single exposure [107].

## 9. Conclusions

The proposition that ultra-processed foods contribute to childhood obesity rests on firmer ground than a decade ago, but the ground is not uniform, and the most defensible reading of the evidence is developmental rather than cumulative.

The mechanistic case is biologically coherent and, in parts, experimentally supported. A degraded food matrix accelerates intake and blunts satiation; additives perturb the assembling gut microbiota and the intestinal barrier; hyperpalatable formulations engage reward circuitry and condition preferences; process-derived chemicals converge with maternal nutrition on the fetal epigenome. Two qualifications run through it. A meaningful part of the matrix effect appears to operate through texture and eating rate, properties not unique to ultra-processed foods, so part of the mechanism lies beyond processing as well as beyond energy density. And the great majority of the experimental evidence is adult or pre-clinical; direct paediatric demonstration is confined to a handful of strands.

The epidemiological association is consistent in direction across settings, with pooled estimates for obesity ranging from a 25% to a twofold increase in odds depending on population and design. It is constrained by exposure misclassification under NOVA, residual socioeconomic and behavioural confounding, reverse causation, weight-patterned dietary misreporting, the near-absence of paediatric trials, and the animal provenance of the mechanistic work.

What the literature permits is a qualified but actionable conclusion. Reducing children’s exposure to discretionary ultra-processed foods, with particular attention to the periconceptional period, infancy, and complementary feeding, and to the textures that drive fast, passive overconsumption, is a reasonable target for obesity prevention, provided that guidance is specific enough to be usable, that infant formula is reported separately from discretionary products (Section 2.1), and that structural measures carry the burden individual counselling cannot. What remains unresolved is the relative contribution of each mechanism, the quantified causal effect of a properly defined exposure, and the window-specificity of these effects in humans. Consistent with the neutral stance taken in Section 2.1, this target is best framed around the specific product characteristics that carry the risk, the degree of processing, the additive load, and the texture that drives passive intake, rather than around the NOVA label itself, which remains too coarse an instrument to guide intervention in the paediatric setting.

The organising claim of this review is that these questions are best approached developmentally. Ultra-processed foods most plausibly exert their influence not by accumulating in the diet of an established organism, but by arriving while the systems that regulate energy balance for a lifetime are still being built. If that is right, then the harm and the opportunity are concentrated in the same narrow window. It must be said plainly that this remains a developmental hypothesis supported mainly by associational human data and by mechanistic work in animals and adults; a causal contribution of ultra-processed foods to childhood adiposity has not been established, and demonstrating it will require the paediatric trials the field still lacks. This distinction between a coherent case and a closed one is worth preserving while we learn which it is.

## Figures and Tables

**Table 1:** Summary of the proposed biological mechanisms linking ultra-processed food intake to childhood adiposity, with the strongest supporting evidence and its predominant evidence base. SCFA, short-chain fatty acids; RCT, randomised controlled trial; PET, positron emission tomography. All entries in this table were compiled by the authors from the primary and review sources cited in Section 4; the table is an original synthesis and is not reproduced from any single published source.

Mechanism	Convergent Process (Figure 2)	Proposed Pathway to Adiposity	Strongest Supporting Evidence	Evidence Base
Food-matrix disruption	Energy-intake regulation (via eating rate)	Softer texture raises eating rate and glycaemic response, weakening satiation	Human trials of texture/energy intake	Adult trials; limited paediatric
Hyperpalatability	Energy-intake regulation (via reward)	Fat–sugar–salt combinations promote passive overconsumption	Sensory and intake studies	Mostly adult
Gut microbiota	Gut-microbiota assembly	Low-fibre, additive-rich diet reshapes community and depletes SCFA	Human and animal microbiome studies; some paediatric cohorts	Mixed; animal-heavy, some paediatric
Intestinal permeability	Intestinal barrier and inflammation	Emulsifiers thin mucus and raise permeability, sustaining low-grade inflammation	Emulsifier RCT (adult) and animal work	Adult RCT; animal; sparse paediatric
Food additives	Gut-microbiota assembly; intestinal barrier	Emulsifiers, sweeteners and thickeners act on gut barrier and microbiota	Mechanistic and exposure studies	Mostly adult/animal
Endocrine-disrupting chemicals	Epigenetic regulation	Phthalates and bisphenols from processing/packaging affect adipogenesis	Biomonitoring cohorts; animal models	Cohort associations; animal mechanism
Eating rate	Energy-intake regulation	Faster ingestion of soft, energy-dense foods increases intake	Texture-based eating-rate trials	Adult trials; some paediatric
Reward signalling	Energy-intake regulation	Engagement of dopaminergic reward may bias food choice	PET/neuroimaging studies	Adult; contested; little paediatric

## Data Availability

No new data were created or analysed in this study. Data sharing is not applicable to this article.
